# Detailed analysis of charge transport in amorphous organic thin layer by multiscale simulation without any adjustable parameters

**DOI:** 10.1038/srep39128

**Published:** 2016-12-21

**Authors:** Hiroki Uratani, Shosei Kubo, Katsuyuki Shizu, Furitsu Suzuki, Tatsuya Fukushima, Hironori Kaji

**Affiliations:** 1 Institute for Chemical Research, Kyoto University, Uji, Kyoto 611-0011, Japan

## Abstract

Hopping-type charge transport in an amorphous thin layer composed of organic molecules is simulated by the combined use of molecular dynamics, quantum chemical, and Monte Carlo calculations. By explicitly considering the molecular structure and the disordered intermolecular packing, we reasonably reproduce the experimental hole and electron mobilities and their applied electric field dependence (Poole–Frenkel behaviour) without using any adjustable parameters. We find that the distribution of the density-of-states originating from the amorphous nature has a significant impact on both the mobilities and Poole–Frenkel behaviour. Detailed analysis is also provided to reveal the molecular-level origin of the charge transport, including the origin of Poole–Frenkel behaviour.

Charge mobility *μ* in organic aggregates is a key factor in understanding the device performance of organic semiconductor devices such as organic light-emitting diodes (OLEDs)^1^,^2^,^3^. Typical organic layers of OLEDs are composed of amorphous thin films. Charge transport in amorphous organic solids is considered to occur through intermolecular charge hopping. Some models have been proposed to describe charge transport in amorphous organic solids. Among these models, the Gaussian disorder model^4^ successfully describes the macroscopic charge transport property. However, the model does not consider actual molecules and it has difficulty in revealing the molecular scale behaviour of carriers; the disorders, which originate from amorphous structures, are used as adjustable parameters to reproduce the experimental mobility data. Although microscopic analyses have also been carried out^5^,^6^,^7^,^8^,^9^,^10^,^11^,^12^,^13^,^14^,^15^,^16^,^17^,^18^,^19^,^20^,^21^,^22^,^23^ and some useful suggestions have been derived, we only have a limited understanding of the nature of charge transport in amorphous organic layers. In these studies, Marcus theory^24^ have frequently been employed to calculate rate constants *k*_*ij*_ of intermolecular charge transfer between molecule *i* and molecule *j*, described as:


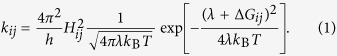


Here, *H*_*ij*_ is the electronic coupling between molecule *i* and *j*. Δ*G*_*ij*_ is the difference of Gibbs free energy between the initial and final states associated with the charge transfer from molecule *i* to *j. λ* is the reorganization energy, *h* is Planck constant, *k*_B_ is Boltzmann constant, and *T* is the absolute temperature.

Our goals are to build a computational model reproducing the experimental *μ* quantitatively, and to gain understanding of the nature of charge transport in amorphous organic layers by analysing the model. We have recently carried out simulations in an amorphous organic solid^23^. The calculated *μ* was overestimated by two orders of magnitude, which also failed to reproduce the experimental observation that *μ* increased as the applied electric field *F* increased.

Here, charge transport process in an amorphous solid of 4,4′-bis(*N*-carbazolyl)-1,1′-biphenyl (CBP) (Supplementary Fig. S1), which is a typical host material for OLEDs, was investigated. In the calculations, we considered the energetic disorder (Fig. 1, see below for details) and intermolecular packing effect on *λ*, both of which were not included in a preceding paper^23^. An amorphous structure containing 4000 CBP molecules was generated using a molecular dynamics (MD) simulation. On the basis of the amorphous structure, *H*_*ij*_ and *λ* were calculated by quantum chemical calculations. Reflecting the amorphous nature, the calculated *H*_*ij*_ values were distributed as shown in Supplementary Figs S2 and S3. The “structural disorder” is often called the “off-diagonal disorder”. In the Gaussian disorder model^4^, material points on a cubic lattice are considered instead of real molecules, and the structural disorder is only an adjustable parameter to describe the experimental results. Here, real molecules are considered; not only the distribution of distances, but also the relative intermolecular orientation and the distribution of frontier orbitals contribute to the distribution of *H*_*ij*_. Examples of the intermolecular packing generated by the above-mentioned MD simulation are shown in Supplementary Fig. S4. From the examples and an intermolecular distance dependence of *H*_*ij*_ (Supplementary Fig. S2), we find that *H*_*ij*_ values are not solely determined by the intermolecular distances. In addition to the structural disorder, molecules (hopping sites) are distributed in energy in amorphous solids (Fig. 1), because different molecules undergo a different intermolecular interaction. The distribution of energy is called the “energetic disorder” or “diagonal disorder”. We calculated the energetic disorder by considering distributed electrostatic interaction with and polarization effect of neighbouring molecules. The energy of a hole/electron on molecule *i*, 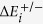, can be written as:


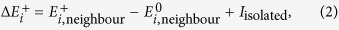



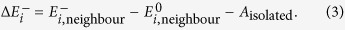


Here, *I*_isolated_ and *A*_isolated_ are the ionization potential and electron affinity of an isolated CBP molecule, respectively. 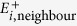, 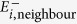, and 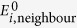 are the sum of electrostatic interaction with and polarization effect of neighbouring molecules, for positively charged, negatively charged, and the neutral state of molecule *i*, respectively. Therefore, Δ*G*_*ij*_ can be expressed as:


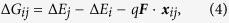


where Δ*E*_*k*_ (*k* = *i,j*) is 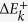 or 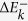 for hole or electron transport, *q* is the charge of the carrier, ***F*** is an externally applied electric field (|***F***| = *F*) and ***x***_*ij*_ is a vector connecting the centre of molecule *i* to the centre of molecule *j* (displacement of the carrier). The calculations in this study show that the distribution of the energy level was Gaussian (see Supplementary Fig. S5) and its sample standard deviation was 0.107 and 0.102 eV for the hole and electron, respectively. *λ* was calculated taking into account the steric influence of neighbouring molecules with the quantum mechanics / molecular mechanics method. Using the calculated *H*_*ij*_, Δ*G*_*ij*_, and *λ, k*_*ij*_ were calculated from equation (1). The charge transport process was simulated using kinetic Monte Carlo method based on the MD-constructed amorphous structure and the *k*_*ij*_, which provided the calculated *μ* (examples of calculated trajectories are shown in Supplementary Fig. S6). To confirm the validity of the calculations, we also performed time-of-flight (TOF) experiments. The experimental data were consistent with those reported previously^20^,^25^,^26^. More details of the computational and the experimental methods are shown later and and in the Supplementary Information.

Figure 2 compares the experimentally-obtained and the calculated *μ* for the hole and electron transport. The experimental *μ* (black filled and black open symbols in Fig. 2) shows a positive linear *F*^1/2^-dependence, often called Poole–Frenkel (PF)-type behaviour, commonly observed in organic amorphous materials^1^,^4^,^27^. Open circles in Fig. 2 consider the intermolecular packing effect in the calculations of *λ* but the energetic disorder is not considered. Here, the *λ* including the intermolecular packing effect in amorphous aggregates is denoted by *λ*_aggr_. The calculated *μ* significantly deviate from the experimental values, being one or two orders of magnitude larger. In addition, the calculated negative *F*^1/2^-dependence is not compatible with the experiments. Open squares in Fig. 2 show the calculated *μ* incorporating the energetic disorder but the intermolecular packing effect on *λ* is not considered (calculated reorganization energy is noted as *λ*_isolated_). By considering the energetic disorder, the experimentally-obtained positive *F*^1/2^-dependence is reproduced. The *μ* is more than one order of magnitude smaller compared with the experiments for electron transport. By incorporating both the energetic disorder and the intermolecular packing effect on *λ*, we obtain the filled squares in Fig. 2. The calculated *μ* showed a better agreement with the experimentally-obtained values. A slightly worse agreement for electron transport is proposed to reflect the difficulty in calculating unoccupied orbitals.

We investigate the origin of the PF behaviour, the positive *F*^1/2^-dependence of *μ*. Figure 2 shows that the PF behaviour is reproduced when the energetic disorder is incorporated in the simulations, but not reproduced when the energetic disorder is not incorporated. Therefore, the energetic disorder plays a crucial role in the PF behaviour. To investigate the mechanism of influence of the energetic disorder on the *F*^1/2^-dependence of *μ*, we calculated the number of hops during the transport *n*, the number of hopping sites used at least once during the transport *ν*, and the mean time required for each individual charge hopping *τ* = *t*/*n (t* is the time required for the charge transport between electrodes), with and without the energetic disorder (see Table 1. The intermolecular packing effect on *λ* is considered in all cases.). When the energetic disorder is included in the simulations, *n* decreases by two orders of magnitude with *F*^1/2^ increasing from 300 V^1/2^cm^−1/2^ to 1300 V^1/2^cm^−1/2^, while *τ* has the same order of magnitude regardless of *F*. Since *μ* = *L*/(*tF*) = *L*/(*nτF*), the PF behaviour is mainly attributed to the decrease in *n* with *F*. Here, *L* is the distance between the electrodes. When *F*^1/2^ = 300 V^1/2^cm^−1/2^ and under the existence of the energetic disorder, *n* is larger than *ν* by two orders of magnitude, indicating that the same sites are repeatedly used. Hence, the charge carriers frequently move back and forth during the charge transport. This “round-trip” of carriers is enhanced by the energetic disorder: without the energetic disorder, *n* has the same order as or is one order of magnitude larger than that of *ν*. Different from the case of *F*^1/2^ = 300 V^1/2^cm^−1/2^, the differences of *n* and *ν* are much smaller when *F*^1/2^ = 1300 V^1/2^cm^−1/2^, regardless of whether the energetic disorder is incorporated or not. In summary, the energetic disorder causes frequent back and forth hopping of carriers and entails large *n* under weak *F*, but not under strong *F*. This results in the PF behaviour. When we compare the simulations with and without the energetic disorder under the same *F*^1/2^, not only *n* but also *τ* become larger by incorporation of the energetic disorder. Both factors affect the reduced *μ* for the case with energetic disorder. When we compare hole and electron transport with the same *F*^1/2^ and energetic disordered state, *n* and *ν* are not significantly different but *τ* is one to two orders of magnitude larger for electron transport. The larger *τ* induces a smaller *μ* for electrons in spite of the maximum *H*_*ij*_ for electron transports (70 meV) being larger than that of hole transports (41 meV). This implies that *μ* is not solely determined by the largest *H*_*ij*_.

These characteristics can be explained at the molecular level. Figure 3 shows the number of hops into site *i* (here, we call it 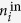) versus their site energies 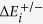. When *F*^1/2^ = 300 V^1/2^cm^−1/2^, 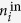 is nearly exponential with the site energies, indicating that the charges mainly use “low-energy” sites (black filled area in Fig. 1) during the transport, that is, charge carriers tend to jump into “low-energy” hopping sites. Here, we note that the “low-energy” site for hole (electron) has a positively (negatively) high energy for the hopping site, as shown in Fig. 1. When the charge carriers escape from the low-energy hopping sites, they tend to back into the low-energy sites owing to the difference of the site energies. Hence, the charge carriers frequently move forward and backward between the low-energy sites and the neighbouring sites under *F*^1/2^ = 300 V^1/2^cm^−1/2^.

The small number of low-energy sites significantly decreases *μ* under weak *F* as demonstrated in the following manner. Figure 4 shows *μ* versus *F*^1/2^ with low-energy 1%, 3%, and 5% of all sites removed. From Fig. 4, *μ* is found to be significantly influenced by the small number (1–5%) of low-energy sites when *F*^1/2^ = 300 V^1/2^cm^−1/2^: it is often considered that low-energy sites trap carriers. Our results suggest that carriers hop back and forth between low-energy sites and the neighbouring sites. The carriers are not frozen in the low-energy sites, but the “round-trip” of carriers consequently consumes much time; therefore, this may also be comprehended as a kind of “trap”.

On the contrary, when *F*^1/2^ = 1300 V^1/2^cm^−1/2^, the correlation between 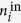 and the site energy is weak [Fig. 3(c)]. Figure 4 clearly shows that *μ* does not change irrespective of the existence of low-energy sites at *F*^1/2^ = 1300 V^1/2^cm^−1/2^, indicating that these sites do not work as “traps”. Charge carriers tend to move along with ***F*** regardless of the energetic disorder.

Next, we discuss the influence of *H*_*ij*_ distribution on *μ. H*_*ij*_ was distributed over the range of 0 to 41 meV and 0 to 70 meV for the hole and electron, respectively (Supplementary Fig. S3). The kinetic Monte Carlo simulations were also performed without molecular pairs with *H*_*ij*_ <  0.1, 1.0, 2.0 and 3.0 meV, and the calculated *μ* are shown in Fig. 5. The molecular pairs with *H*_*ij*_ < 0.1 meV do not affect the charge transports (open circles in Fig. 5 are overlapped with filled squares). However, the contributions of the pairs with *H*_*ij*_ <  1.0, 2.0 and 3.0 meV are significant, especially at strong *F*. When the pairs with *H*_*ij*_ < 3.0 meV are removed (downward open triangles in Fig. 5), *μ* decreases by three or four orders of magnitude. This result clearly shows that the pairs with *H*_*ij*_ < 3.0 meV are frequently used as charge hopping sites although the *H*_*ij*_ values are much smaller than the maximum *H*_*ij*_ (41 meV and 70 meV for hole and electron hopping, respectively). Hence, the decrease in *μ* by removing pairs with *H*_*ij*_ < 1.0–3.0 meV is attributed to the change in charge transport paths. This result indicates that in amorphous aggregates, weakly coupled pairs significantly contribute to *μ* by forming effective charge transport paths especially at strong *F*.

From the above discussion, in amorphous thin layers, *μ* is not simply determined by the maximum *H*_*ij*_ and therefore the maximum *k*_*ij*_, although the values of *μ* are often discussed by the *H_ij_* or *k*_*ij*_. This feature is different from that of crystalline solids. Thus, consideration of different hierarchical structures is crucial for the understanding of *μ* in amorphous organic aggregates.

In conclusion, *μ* was quantitatively reproduced without any adjustable parameters, by taking into account both the disorder of the energy level of the hopping sites and the disordered structure. The PF behaviour was also reproduced, and its origin was attributed to the influence of the energetic disorder on the charge transport trajectory: under weak *F*, charge carriers go back and forth owing to the difference of the site energies; however, under strong *F*, charge carriers tend to move simply along ***F***. In amorphous organic solids, molecular pairs with relatively small *H*_*ij*_ are found to largely contribute to *μ*, indicating that they form effective paths for charge transport.

## Methods

### Simulation of charge transport process

Simulations of the charge transport process for amorphous structure containing 100, 1000, 4000, and 8000 molecules of CBP were performed. The results for the 4000 CBP system are provided in the main text. The MD simulation was performed on LAMMPS program package^28^. Density functional theory and extended Hückel calculations were carried out using the Gaussian 09 program package^29^. Kinetic Monte Carlo simulations were performed using our in-house program. Details of methodology are described in Supplementary Information.

### Experimental TOF measurement

*μ* was experimentally measured for a CBP thin film using TOF apparatus (TOF-401-3, Sumitomo Heavy Industries Advanced Machinery Co., Ltd., Japan). A TOF sample with the structure of ITO (50 nm)/CBP (3.9 μm)/Al (20 nm) was fabricated by vacuum-deposition. A N_2_ gas laser (KEC-150, Usho Optical Systems Co., Ltd., Japan) with a wavelength of 337 nm was used to generate the photocarriers.

## Additional Information

**How to cite this article**: Uratani, H. *et al*. Detailed analysis of charge transport in amorphous organic thin layer by multiscale simulation without any adjustable parameters. *Sci. Rep.* **6**, 39128; doi: 10.1038/srep39128 (2016).

**Publisher's note:** Springer Nature remains neutral with regard to jurisdictional claims in published maps and institutional affiliations.

## Supplementary Material

Supplementary Information

## Figures and Tables

**Figure 1:**
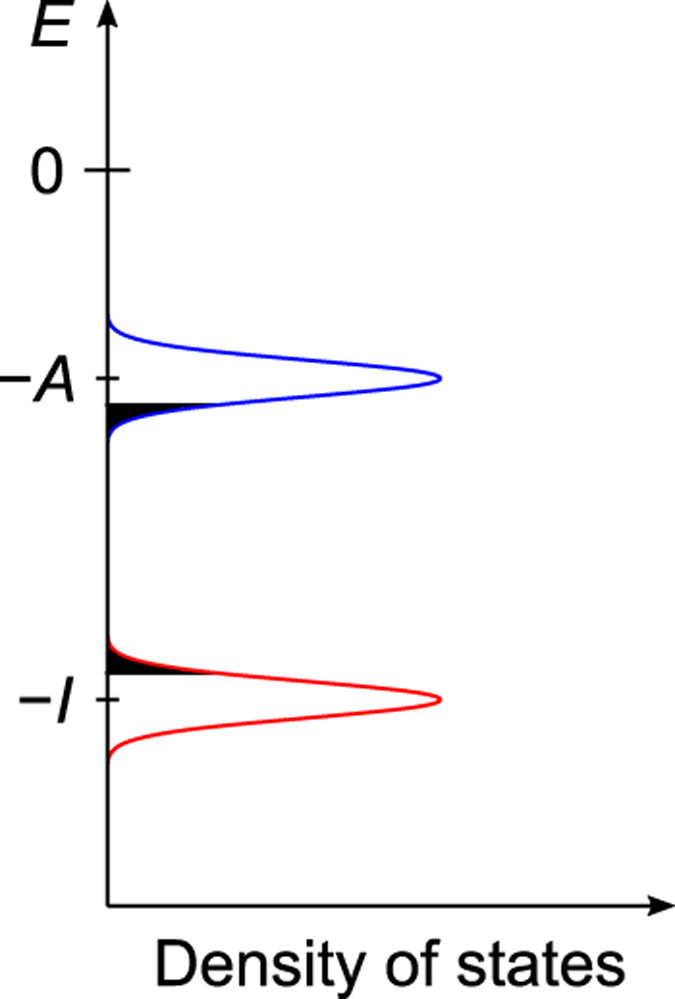
Schematic illustration of energetic disorder. *A* and *I* are electron affinity and ionization potential in amorphous structure, respectively.

**Figure 2:**
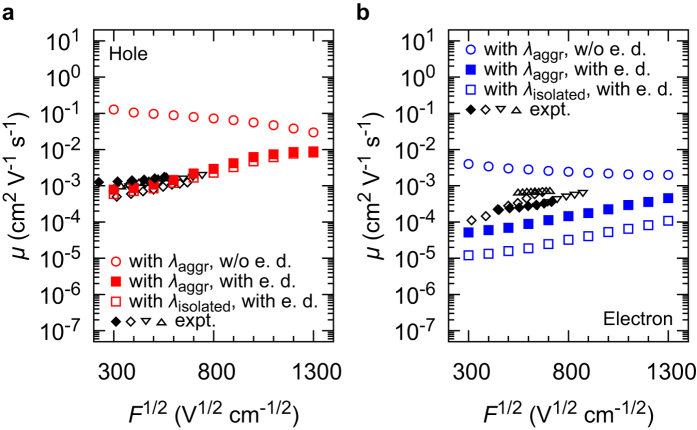
Calculated and experimental *μ* in an amorphous CBP film. (**a**) For hole transport. (**b**) For electron transport. Black filled diamonds: measured by TOF experiments. Black open diamonds, downward triangles, and upward triangles: experimental data from Refs 20, 25 and 26, respectively. Open circles: calculated with *λ*_aggr_ and without energetic disorder (e. d.). Filled squares: calculated with *λ*_aggr_ and with e. d. Open squares: calculated with *λ*_isolated_ and with e. d.

**Figure 3:**
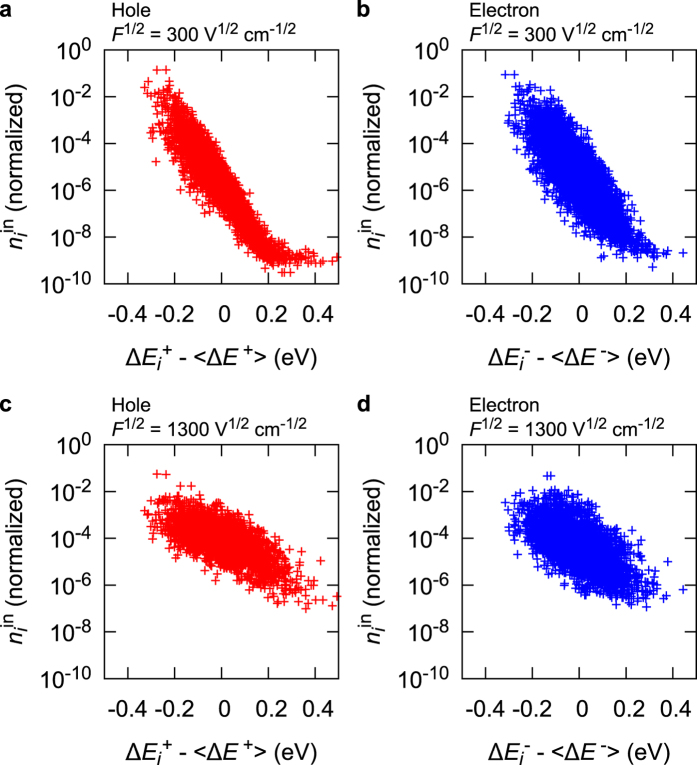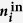 versus their site energies. (**a**), (**b**) For hole and electron under *F*^1/2^ = 300 V^1/2^cm^−1/2^, respectively. (**c**), (**d**) For hole and electron under *F*^1/2^ = 1300 V^1/2^cm^−1/2^, respectively. 〈Δ*E*^+^〉 and 〈Δ*E*^−^〉 are average values of 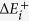 and 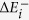, respectively.

**Figure 4:**
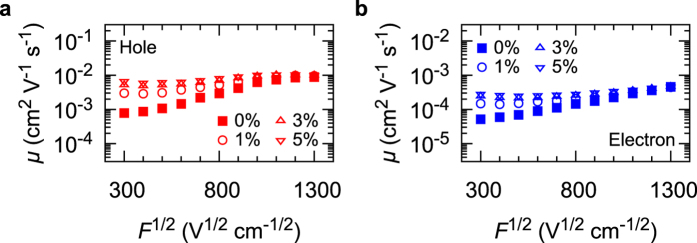
*μ* calculated without low-energy sites. (**a**) For hole. (**b**) For electron. Open circles, open upward triangles, and open downward triangles: 1%, 3%, and 5% of low-energy sites removed, respectively. Filled squares: calculated *μ* with all hopping sites.

**Figure 5:**
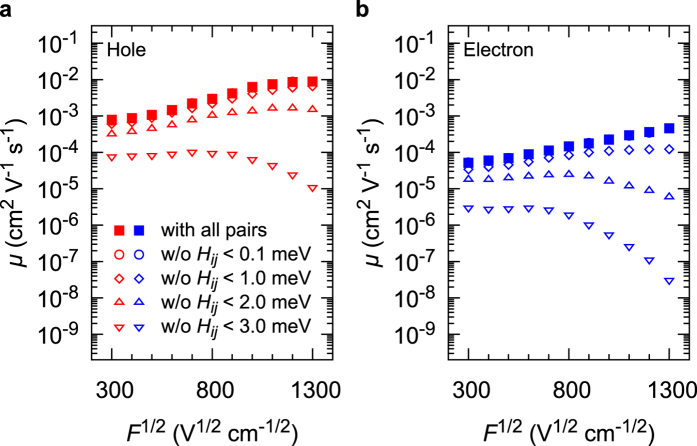
*μ* calculated under various conditions. (**a**) For hole. (**b**) For electron. Filled squares: all the pairs are used. Open circles (overlapped with filled squares): pairs with *H*_*ij*_ < 0.1 meV are removed. Open diamonds: pairs with *H*_*ij*_ < 1.0 meV are removed. Upward open triangles: pairs with *H*_*ij*_ < 2.0 meV are removed. Downward open triangles: pairs with *H*_*ij*_ < 3.0 meV are removed.

**Table 1:** The number of hops *n*, the number of used hopping sites *ν*, and the mean time required for each individual charge hopping *τ* = *t*/*n*.

Carrier	*F*^1/2^ (V^1/2^cm^−1/2^)	*n*	*ν*	*τ* (ps)
Hole (with energetic disorder)	300	220,324	1,265	2.6
Hole (with energetic disorder)	1300	1,022	266	4.3
Electron (with energetic disorder)	300	128,683	1,176	58.8
Electron (with energetic disorder)	1300	1,136	185	74.9
Hole (without energetic disorder)	300	4,729	1,257	0.4
Hole (without energetic disorder)	1300	405	235	0.9
Electron (without energetic disorder)	300	12,584	1,325	5.2
Electron (without energetic disorder)	1300	507	179	19.4

Intermolecular packing effect on *λ* is considered in all cases.

All of the values are the averages of 10,000 trials of the kinetic Monte Carlo simulation.
