# Supplementary material for: Detailed analysis of charge transport in amorphous organic thin layer by multiscale simulation without any adjustable parameters
Source: Sci Rep. 2016 Dec 21;6:39128. doi: 10.1038/srep39128 (PMC5175281; doi:10.1038/srep39128)
Supplement: Supplementary Information [file srep39128-s1.pdf]

Supplementary Information for: Detailed analysis of charge transport in amorphous organic thin layer by multiscale simulation without any adjustable parameters

Hiroki Uratani, Shosei Kubo, Katsuyuki Shizu, Furitsu Suzuki, Tatsuya Fukushima, and Hironori Kaji*

Institute for Chemical Research, Kyoto University, Uji, Kyoto 611-0011, Japan
*kaji@scl.kyoto-u.ac.jp

1 Details of methodology

Simulations of the charge transport process for amorphous structure containing 100, 1000, 4000, and 8000 molecules of 4,4'-bis(*N*-carbazolyl)-1,1'-biphenyl (CBP) were performed. Molecular structure of CBP is shown in Fig. S1. Here, the methodology for the 4000 CBP system is described. We performed the simulations also for the other systems in the same manner as performed for the 4000 CBP system. The MD simulation was performed on LAMMPS program package[1]. Density functional theory (DFT) and extended Hückel calculations were carried out using the Gaussian 09 program package[2]. Kinetic Monte Carlo simulations were performed using our in-house program.

1.1 MD simulation

The amorphous structure containing 4000 CBP molecules was generated with MD simulation. The Dreiding force field[3] was used, the bond length parameters were modified to reproduce the optimized molecular structure obtained with DFT. The DFT-optimized bond angles were reproduced by the MD simulation. The cubic cell was generated and periodic boundary condition was applied. A Lennard-Jones $(12,6)$ potential was used as a model for the van der Waals interaction with tail correction[4], which is a correction for thermodynamic quantities that takes into account the homogeneous long range van der Waals inter-

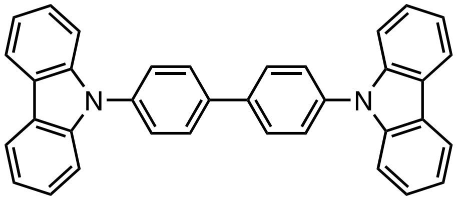

Figure S1. **Structural formula of CBP.**

action. Particle–particle-particle–mesh method[5] was used to calculate Coulombic interaction between atoms. To determine the atomic charges, we performed DFT calculation on an optimized structure of isolated CBP molecule and then used the Merz–Singh–Kollman scheme[6], which is a method for calculating atomic charges to fit the electrostatic potential on molecular surfaces.

As an initial structure of the MD simulation, the DFT-optimized molecules were randomly placed in a large cubic cell. To mimic the vapor deposition process, the MD simulation was performed in a NVT ensemble under 573 K for 10 ps followed by a simulation in a NPT ensemble under 298 K and 1.0×10^{-4} Pa for 1.0 ns using a Nosé–Hoover thermostat and barostat[7,8]. During the simulation in the NPT ensemble, the simulation cell shrank and finally reached a constant volume. We obtained the cubic cell of the condensed structure with a side length of 14.55 nm and the density of the generated structure was calculated to be 1.04 g cm^{-3}. Finally, to eliminate the deviation from the stable structure originating from molecular vibration, geometry optimization was performed.

1.2 Reorganization energy (λ)

Structure optimizations and the calculations of λ were performed by DFT calculations [Becke three-parameter Lee–Yang–Parr (B3LYP) functional[9] and the 6-31G(d) basis set]. λ is described as $\lambda = \lambda_1 + \lambda_2$. λ_1 and λ_2 are described as:

$$\lambda_1 = E_{\mathrm{n}}^{\mathrm{c}} - E_{\mathrm{c}}^{\mathrm{c}}, \tag{1}$$

$$\lambda_2 = E_{\mathrm{c}}^{\mathrm{n}} - E_{\mathrm{n}}^{\mathrm{n}}, \tag{2}$$

where $E_{\mathrm{n}}^{\mathrm{c}}$, $E_{\mathrm{c}}^{\mathrm{c}}$, $E_{\mathrm{c}}^{\mathrm{n}}$, and $E_{\mathrm{n}}^{\mathrm{n}}$ are the molecular energy of the charged state in the neutral optimized geometry, the charged state in the charged optimized geometry, the neutral state in the charged optimized geometry, and the neutral state in the neutral optimized geometry, respectively[10]. In most cases, λ is calculated for an isolated molecule. However, interactions with neighbouring molecules in the aggregate structure are considered to affect λ. Here, we introduced a quantum mechanics / molecular mechanics (QM/MM) approach[11] to quantify such environmental influences. The λ including the influences is denoted by λ_{aggr}. For 45 randomly sampled molecules (out of 4000 molecules in the aggregate structure), we calculated λ_{aggr} by the QM/MM method. DFT and the Dreiding force field were used for the QM and MM regions,

respectively. The neighbouring molecules within 30 Å were included and their geometries were frozen during the calculation. Partial charges of the atoms in the MM region were incorporated into the QM Hamiltonian. The partial charges were set to be same values as those used in MD calculation. The calculated average value of λ_{aggr} was 0.105 ± 0.009 eV for holes and 0.373 ± 0.027 eV for electrons (95% confidence interval), respectively. We also calculated the reorganization energy for the optimized isolated CBP molecule, $\lambda_{\text{isolated}}$. $\lambda_{\text{isolated}}$ (0.129 eV and 0.516 eV for hole and electron, respectively) is larger than λ_{aggr}, owing to the absence of the steric effect from neighbouring molecules.

1.3 Electronic coupling (H_{ij})

H_{ij} was calculated using the extended Hückel method as described in previous work[10,12,13]. Calculation was performed for all pairs with a centre-to-centre distance within 25 Å (167,993 pairs). The cutoff distance (25 Å) is validated by H_{ij} versus the intermolecular distance plots (Fig. S2), which show that the molecular pairs with a centre-to-centre distance longer than 25 Å have a negligibly small H_{ij} ($<10^{-4}$ meV). Figure S3 shows the distributions of the calculated H_{ij} for hole and electron. Some examples of the intermolecular packings are shown in Fig. S4.

1.4 Energetic disorder

We calculated the energetic disorder by considering distributed electrostatic interaction with and polarization effect of neighbouring molecules. $E^{0/+/-}_{i,\text{neighbour}}$, the sum of the two terms, is then:

$$E^{0/+/-}_{i,\text{neighbour}} = E^{0/+/-}_{i,\text{charge-charge}} + E^{0/+/-}_{i,\text{charge-dipole}}. \tag{3}$$

Here, the superscripts 0, +, and − represent neutral, positively charged, and negatively charged states of molecule i, respectively. $E^{0/+/-}_{i,\text{charge-charge}}$, defined by:

$$E^{0/+/-}_{i,\text{charge-charge}} = \sum_{j}\sum_{k}\sum_{k'} \frac{q_{i,k}q_{j,k'}}{4\pi\varepsilon_0}\frac{1}{|\boldsymbol{r}_{ij,kk'}|}, \tag{4}$$

is an electrostatic interaction energy between the atomic partial charges of molecule i and neighbouring molecules, where $q_{i,k}$ and $q_{j,k'}$ are an atomic partial charge of atom k in molecule i and that of atom k' in molecule j, respectively, and $\boldsymbol{r}_{ij,kk'}$ is a vector connecting atom k in molecule i to atom k' in molecule j.

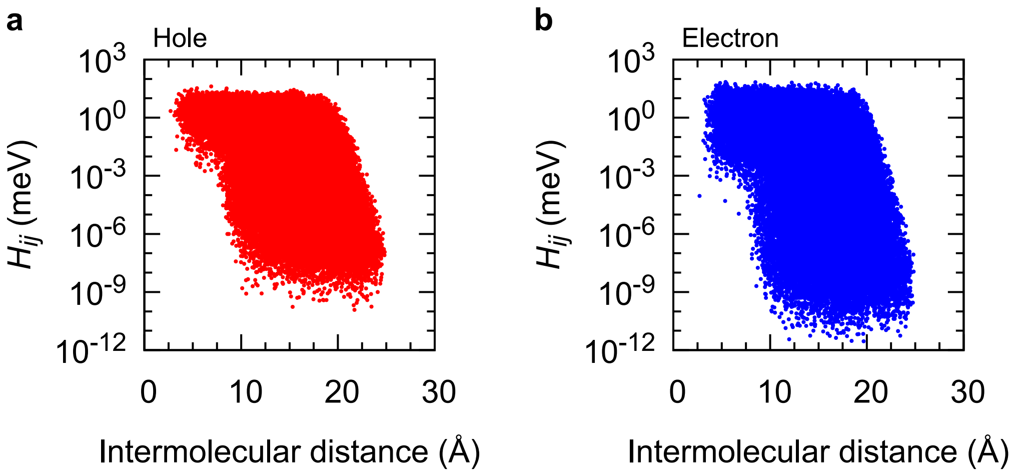

Figure S2. **H_{ij} versus intermolecular (centre-to-centre) distance.** (a) For hole. (b) For electron.

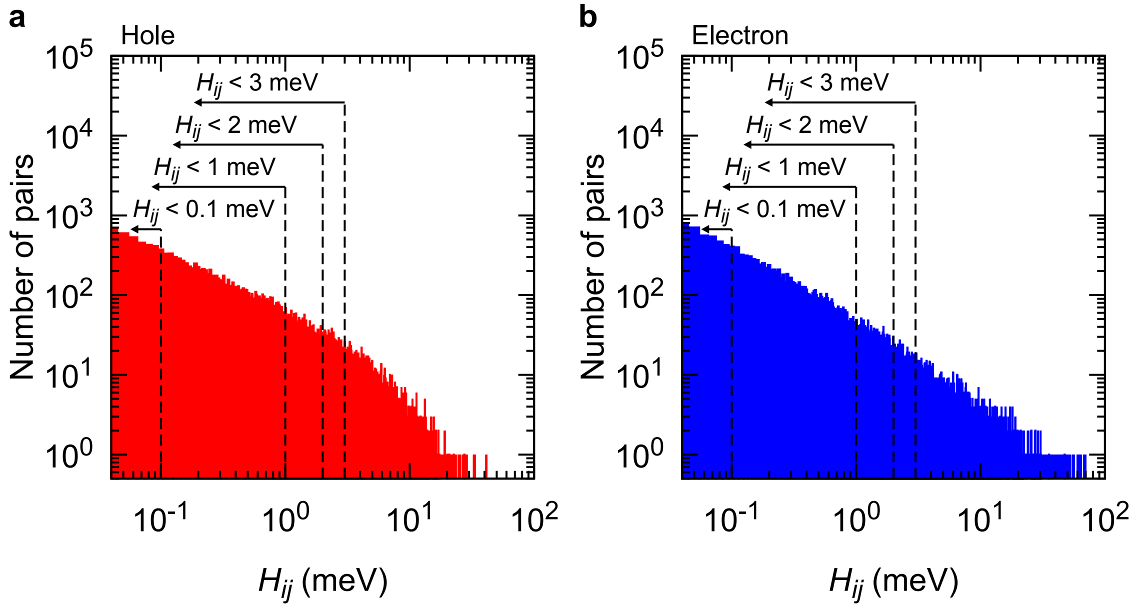

Figure S3. **Distributions of H_{ij} for amorphous CBP.** (a) For hole. (b) For electron.

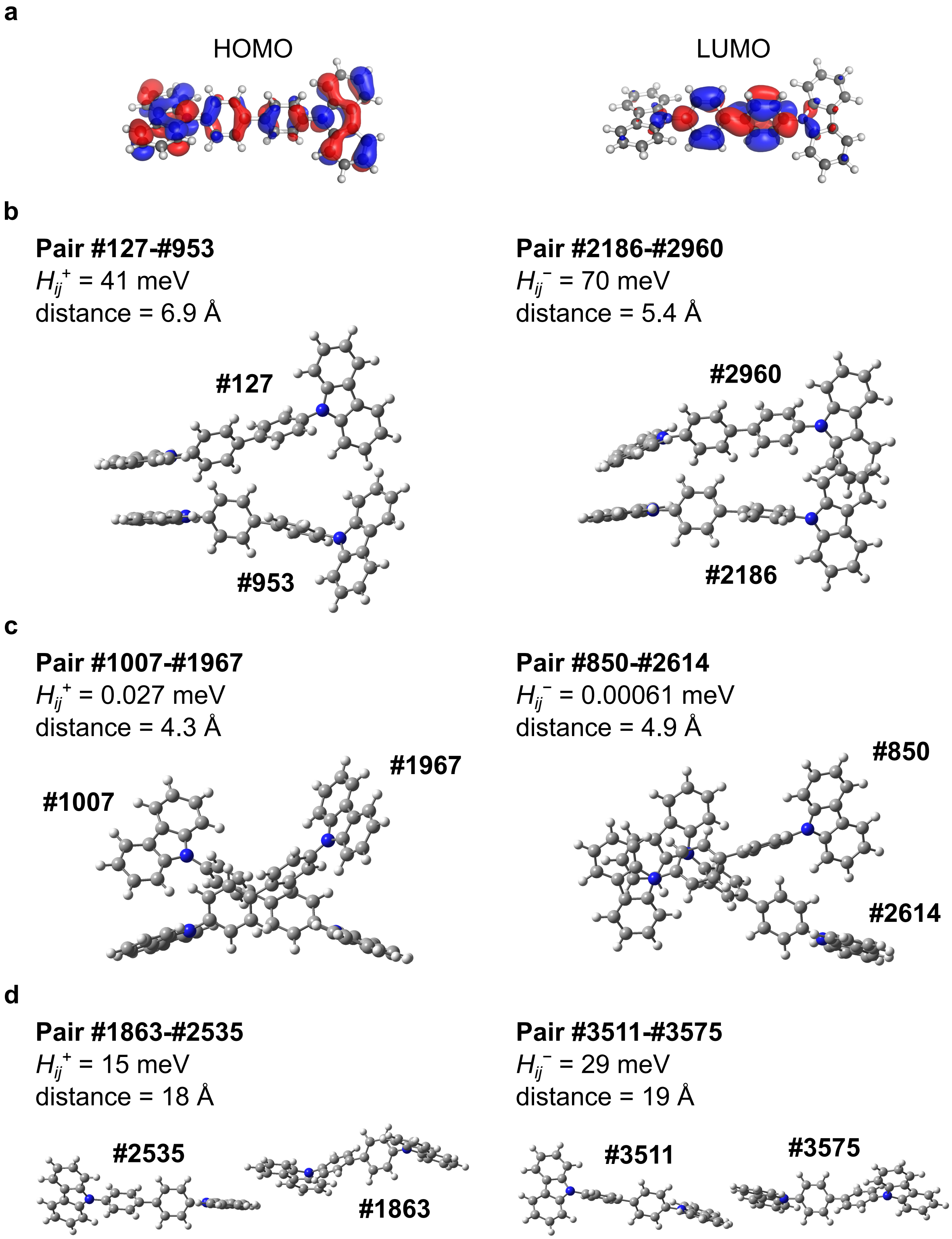

Figure S4. **Frontier orbitals and examples of intermolecular packings.** H_{ij}^{+} and H_{ij}^{-} are H_{ij} for hole and electron transport, respectively. (a) HOMO and LUMO of isolated CBP. (b) Molecular pairs which have largest H_{ij}. (c) Molecular pairs which have small H_{ij} in spite of a short distance. (d) Molecular pairs which have large H_{ij} in spite of a long distance. Note that the distributions of frontier orbitals are significantly depend on the conformation of the molecule in the aggregate structure.

Atomic partial charges of charged and neutral molecules were obtained by DFT calculation and the Merz–Singh–Kollman scheme (same method as used for the MD simulation). $E^{0/+/-}_{i,\text{charge-charge}}$ was calculated taking into account neighbouring molecules with a centre-to-centre distance within 30 Å. $E^{0/+/-}_{i,\text{charge-dipole}}$ is a polarization effect of neighbouring molecules, which effectively moderates the interaction between atomic partial charges. This effect can be regarded as the interaction between atomic partial charges in molecule i and induced dipole moments on atoms in neighbouring molecules. Hence, $E^{0/+/-}_{i,\text{charge-dipole}}$ can be approximately written as:

$$E^{0/+/-}_{i,\text{charge-dipole}} = \sum_j \sum_k \sum_{k'} -\frac{q_{i,k}}{4\pi\varepsilon_0} \frac{\boldsymbol{\mu}_{j,k'} \cdot \boldsymbol{r}_{ij,kk'}}{|\boldsymbol{r}_{ij,kk'}|^3}, \tag{5}$$

where $\boldsymbol{\mu}_{j,k'}$ is an induced dipole moment on atom k' in neighbouring molecule j. $\boldsymbol{\mu}_{j,k'}$ can be described as $\boldsymbol{\mu}_{j,k'} = \alpha_{j,k'}\boldsymbol{E}$, where $\alpha_{j,k'}$ is an atomic polarizability and $\boldsymbol{E}$ is an electric field at the position of atom k' in molecule j caused by the atomic partial charges in molecule i and its neighbours within 30 Å other than molecule j. We used atomic polarizability values implemented in the AMOEBA polarizable force field[14]. By equations (3), (4), and (5), we can determine the energies of the positively charged state ($E^+_{i,\text{neighbour}}$), negatively charged state ($E^-_{i,\text{neighbour}}$), and neutral state ($E^0_{i,\text{neighbour}}$) for molecule i. The energy level of a hole/electron on molecule i, $\Delta E^{+/-}_i$, can be written as:

$$\Delta E^+_i = E^+_{i,\text{neighbour}} - E^0_{i,\text{neighbour}} + I_\text{isolated}, \tag{6}$$

$$\Delta E^-_i = E^-_{i,\text{neighbour}} - E^0_{i,\text{neighbour}} - A_\text{isolated}, \tag{7}$$

where I_isolated and A_isolated are the ionization potential and electron affinity of an isolated CBP molecule, respectively. Therefore, the difference of the Gibbs free energy associated with the hole/electron transfer from molecule i to j, $\Delta G^{+/-}_{ij}$, can be expressed as:

$$\Delta G^{+/-}_{ij} = \Delta E^{+/-}_j - \Delta E^{+/-}_i - q\boldsymbol{F} \cdot \boldsymbol{x}_{ij}, \tag{8}$$

where q is the charge of the carrier, $\boldsymbol{F}$ is an externally applied electric field ($|\boldsymbol{F}| = F$) and $\boldsymbol{x}_{ij}$ is a vector connecting the centre of molecule i to the centre of molecule j (displacement of the carrier). The distri-

bution of the energy level obtained in this work was Gaussian (Fig. S5) and its sample standard deviation was 0.107 eV and 0.102 eV for the hole and electron, respectively.

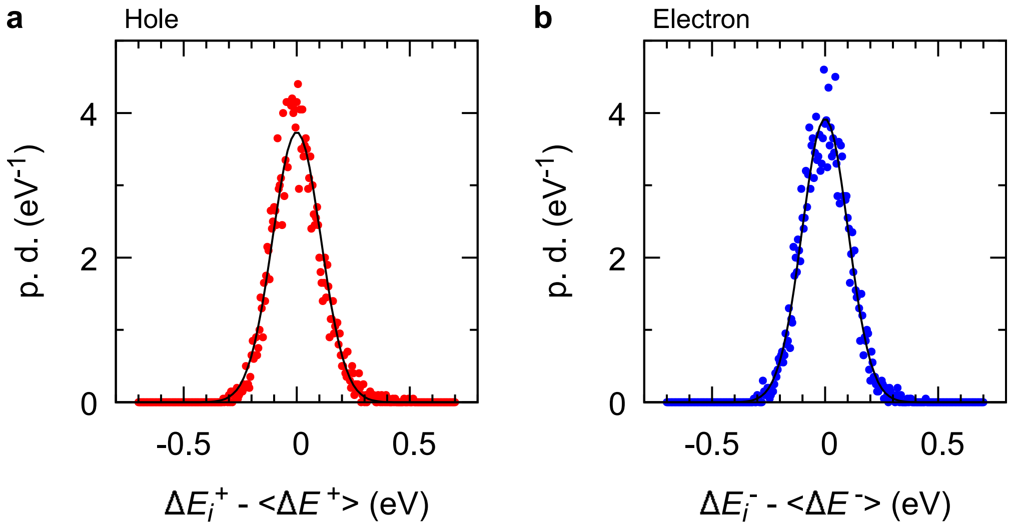

Figure S5. Probability densities (p. d.) of the deviation from the average energy level. (a) For hole. (b) For electron. Solid lines show the Gaussian probability density function, which is zero-centred and whose standard deviation is set to be the sample standard deviation of the calculated values.

1.5 Kinetic Monte Carlo simulation

Using rate constants for the charge transfer from molecule i to j, $k_{ij}^{+/-}$, calculated with the Marcus equation (the superscript $+/-$ denotes that the quantities are for hole/electron transport):

$$k_{ij}^{+/-} = \frac{4\pi^2}{h}(H_{ij}^{+/-})^2 \frac{1}{\sqrt{4\pi\lambda^{+/-}k_\mathrm{B}T}} \exp\left[-\frac{(\lambda^{+/-}+\Delta G_{ij}^{+/-})^2}{4\lambda^{+/-}k_\mathrm{B}T}\right], \tag{9}$$

a kinetic Monte Carlo calculation was performed to simulate the experimental time-of-flight (TOF) measurements. The simulation was performed under 298 K until the charge moved a distance L along $\boldsymbol{F}$, with a periodic boundary condition for each axes. We set L to be 100 nm. 10,000 trials were performed to obtain the TOF transient and calculate the travelling time t. Examples of the simulated trajectories are shown in Fig. S6. μ was calculated from $\mu = L/(tF)$ for the three orthogonal directions, x-, y-, and z-axes. The calculated μ for the three directions is shown in Fig. S7. An anisotropy of μ was observed

for the MD-constructed amorphous structures which consist of 100 or 1000 CBP molecules. For the structures containing 4000 or 8000 CBP molecules, the anisotropy almost disappeared, and the averaged values of μ calculated for the three directions were consistent for 4000 and 8000 systems. The averaged μ are provided in the main text.

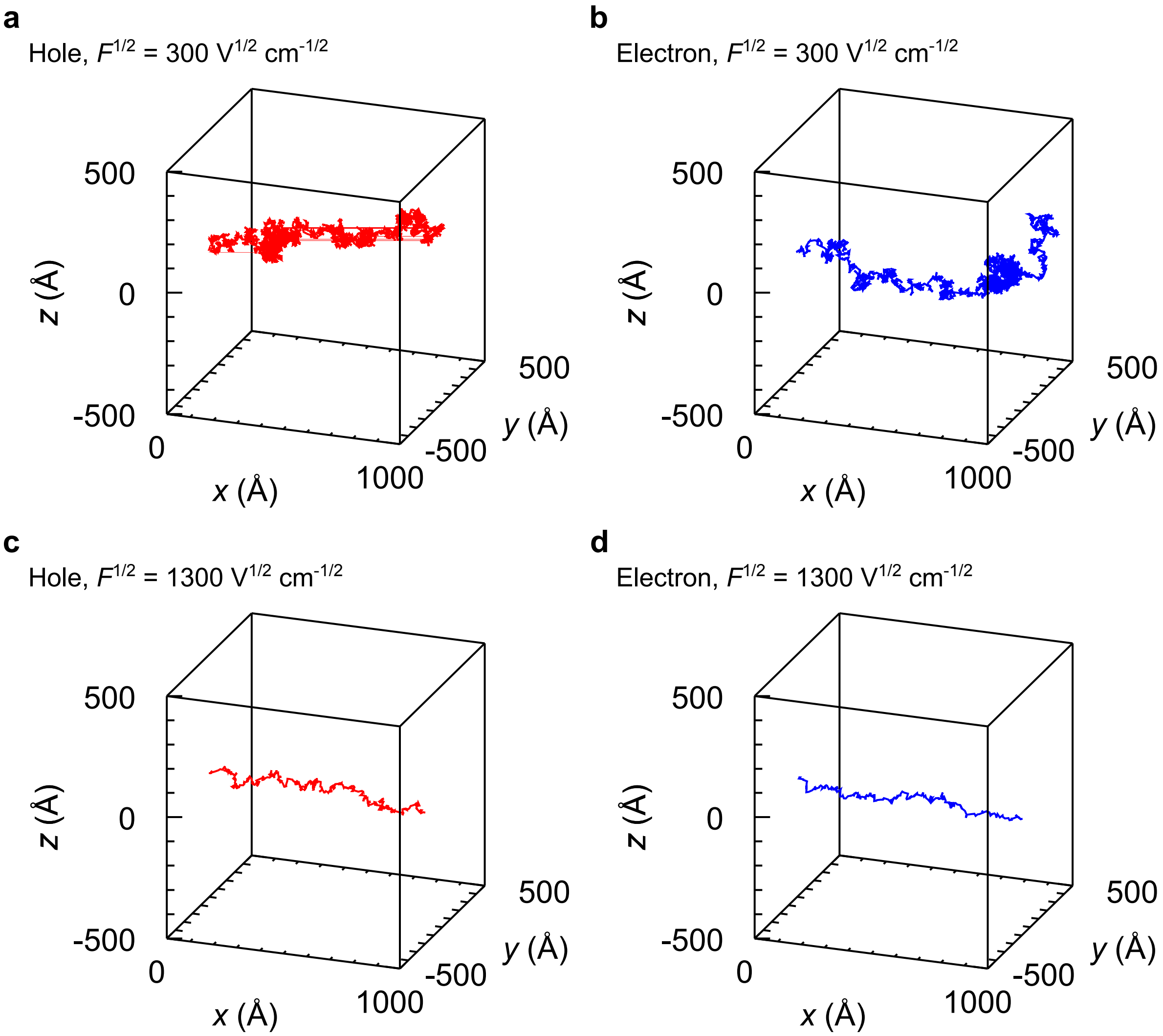

Figure S6. Examples of the simulated transport trajectories. The ***F*** is applied along the *x*-axis. (a), (b) For hole and electron transport under $F^{1/2} = 300\,\mathrm{V}^{1/2}\,\mathrm{cm}^{-1/2}$, respectively. (c), (d) For hole and electron transport under $F^{1/2} = 1300\,\mathrm{V}^{1/2}\,\mathrm{cm}^{-1/2}$, respectively.

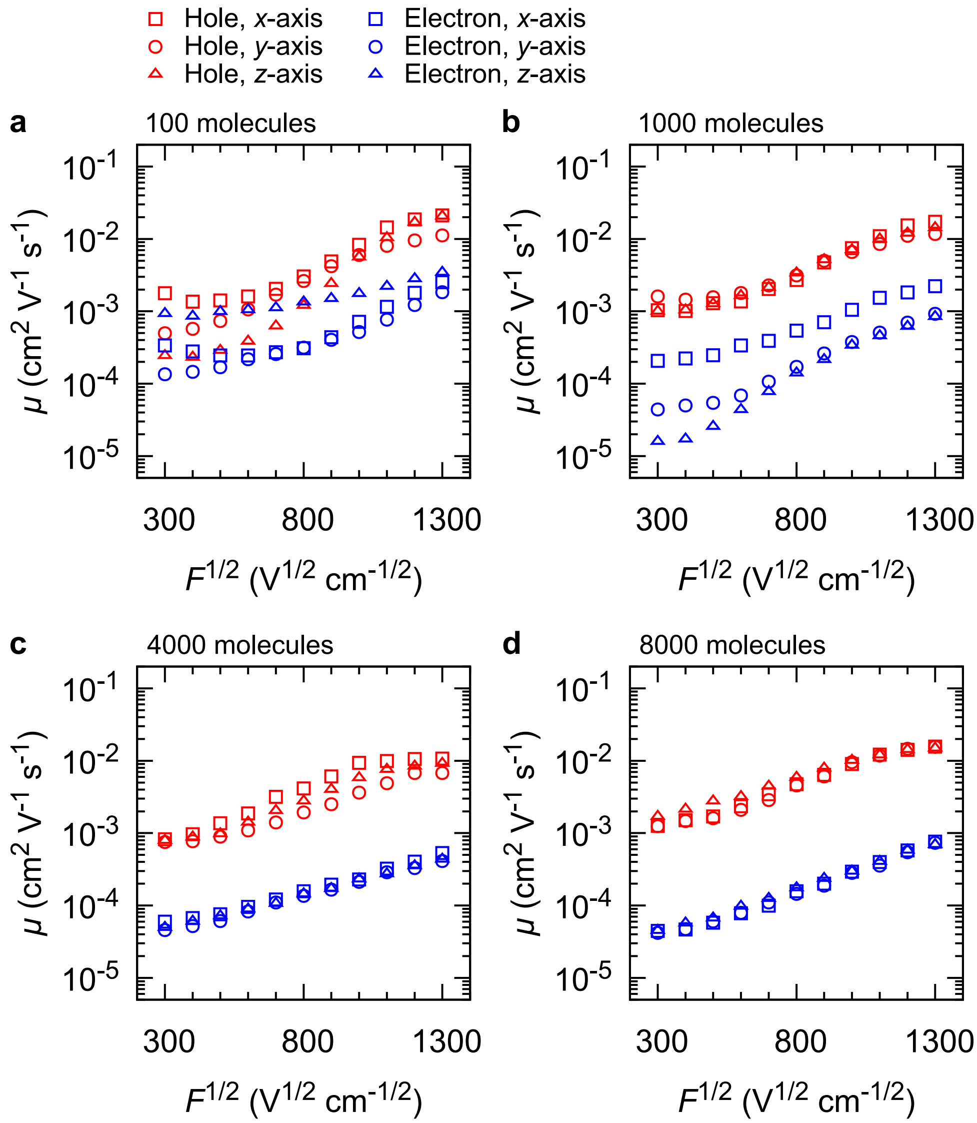

Figure S7. Calculated μ for hole and electron transport with various size of system. μ was calculated for three orthogonal axes with (a) 100, (b) 1000, (c) 4000, and (d) 8000 CBP systems.

References

1. Plimpton, S. Fast parallel algorithms for short-range molecular dynamics. *J. Comput. Phys.* **117**, 1–19 (1995).

2. Frisch, M. J. *et al.* Gaussian 09, Revision C.01 and D.01. Gaussian, Inc., Wallingford CT, 2009.

3. Mayo, S. L., Olafson, B. D. & Goddard, W. A. Dreiding: a generic force field for molecular simulations. *J. Phys. Chem.* **94**, 8897–8909 (1990).

4. Sun, H. COMPASS: An ab initio force-field optimized for condensed-phase applications—overview with details on alkane and benzene compounds. *J. Phys. Chem. B* **102**, 7338–7364 (1998).

5. Hockney, R. W. & Eastwood, J. W. *Computer simulation using particles* (CRC Press, 1988).

6. Singh, U. C. & Kollman, P. A. An approach to computing electrostatic charges for molecules. *J. Comput. Chem.* **5**, 129–145 (1984).

7. Nosé, S. A unified formulation of the constant temperature molecular dynamics methods. *J. Chem. Phys.* **81**, 511–519 (1984).

8. Hoover, W. G. Canonical dynamics: Equilibrium phase-space distributions. *Phys. Rev. A* **31**, 1695–1697 (1985).

9. Becke, A. D. Density-functional thermochemistry. III. the role of exact exchange. *J. Chem. Phys.* **98**, 5648–5652 (1993).

10. Yamada, T., Sato, T., Tanaka, K. & Kaji, H. Percolation paths for charge transports in N,N'-diphenyl-N,N'-di(m-tolyl)benzidine (TPD). *Org. Electron.* **11**, 255–265 (2010).

11. Li, H., Duan, L., Zhang, D. & Qiu, Y. Influence of molecular packing on intramolecular reorganization energy: A case study of small molecules. *J. Phys. Chem. C* **118**, 14848–14852 (2014).

12. Yamada, T. *et al.* Revealing bipolar charge-transport property of 4,4'-N,N'-dicarbazolylbiphenyl (CBP) by quantum chemical calculations. *Org. Electron.* **12**, 169–178 (2011).

13. Suzuki, F. *et al.* Multiscale simulation of charge transport in a host material, N,N'-dicarbazole-3,5-benzene (mCP), for organic light-emitting diodes. *J. Mater. Chem. C* **3**, 5549–5555 (2015).

14. Ponder, J. W. *et al.* Current status of the AMOEBA polarizable force field. *J. Phys. Chem. B* **114**, 2549–2564 (2010).
